# Structural Determinants of Isoform Selectivity in PI3K Inhibitors

**DOI:** 10.3390/biom9030082

**Published:** 2019-02-26

**Authors:** Michelle S. Miller, Philip E. Thompson, Sandra B. Gabelli

**Affiliations:** 1 Department of Oncology, Johns Hopkins University School of Medicine, Baltimore, MD 21205, USA; michelle.miller@jhmi.edu; 2 Medicinal Chemistry, Monash Institute of Pharmaceutical Sciences, Parkville, VIC 3052, Australia; philip.thompson@monash.edu; 3 Departments of Medicine, Biophysics and Biophysical Chemistry, Johns Hopkins University School of Medicine, Baltimore, MD 21205, USA

**Keywords:** PIK3CA, isoform selectivity, p110, p85, phosphatidylinositol 3-kinase, PI3Kβ, PI3Kα, PI3Kγ, PI3Kδ

## Abstract

Phosphatidylinositol 3-kinases (PI3Ks) are important therapeutic targets for the treatment of cancer, thrombosis, and inflammatory and immune diseases. The four highly homologous Class I isoforms, PI3Kα, PI3Kβ, PI3Kγ and PI3Kδ have unique, non-redundant physiological roles and as such, isoform selectivity has been a key consideration driving inhibitor design and development. In this review, we discuss the structural biology of PI3Ks and how our growing knowledge of structure has influenced the medicinal chemistry of PI3K inhibitors. We present an analysis of the available structure-selectivity-activity relationship data to highlight key insights into how the various regions of the PI3K binding site influence isoform selectivity. The picture that emerges is one that is far from simple and emphasizes the complex nature of protein-inhibitor binding, involving protein flexibility, energetics, water networks and interactions with non-conserved residues.

## 1. Introduction

With more than 500 members in the human kinome, kinases are the most common family of enzymatic drug targets for the treatment of cancer and other diseases [1]. However, with so many diverse members and functions, inhibitor selectivity is both necessary to dissect individual biological functions, and a key safety concern in translating these inhibitors to the clinic. This is perhaps most difficult to achieve among isoforms of related kinases, which contain highly conserved ATP binding sites. Phosphatidylinositol 3-kinases (PI3Ks), and in particular, the four members of Class I, are such a family. PI3Ks are a family of lipid kinases that phosphorylate phosphatidylinositides at the 3’ position of the inositol ring. They have been divided into three classes based on their substrate specificity and sequence homology. Class I PI3Ks, which will form the focus of this review, phosphorylate phosphatidylinositol-4,5-bisphosphate (PIP_2_) downstream of either receptor tyrosine kinases or G-protein coupled receptors to form the second messenger phosphatidylinositol-3,4,5-trisphosphate (PIP_3_), which signals for increased cell growth, metabolism, and cell-cycle progression [2]. Class I consists of four family members, each of which forms a heterodimer between a catalytic subunit, p110, and a regulatory subunit. The family is further subdivided into Class IA, where p110α, p110β and p110δ form heterodimers with the p85 family of regulatory subunits [3,4], and Class IB, where p110γ is the sole member and forms a heterodimer with either the p87 or p101 regulatory subunit [5,6,7].

Each isoform has been shown to have overlapping, but non-redundant physiological roles [8]. PI3Kα and β are both ubiquitously expressed. PI3Kα plays a key role in glucose homeostasis and insulin signaling [9,10] and is involved in driving myocardial growth via PIP_3_ dependent pathways [11,12,13,14]. PI3Kβ, in contrast, has been shown to regulate the activity of the platelet integrin α_IIb_β_3_ in the context of platelet adhesion and aggregation [15,16,17]. As such PI3Kβ is under investigation as a novel treatment for thrombosis, and the first in-human trials have shown promising results [16]. PI3Kγ and δ have more restricted expression, largely limited to the hematopoietic system. They both play important, non-redundant roles in the immune system, and so are both under consideration as immune modulatory targets [18,19,20]. PI3Kγ inhibition is being pursued for rheumatoid arthritis and asthma [20,21,22], and PI3Kδ for activated PI3Kδ syndrome (APDS) [23,24,25,26,27].

Their diverse functions notwithstanding, PI3Ks are perhaps most well-known as oncology targets. The PI3K pathway is one of the most frequently dysregulated in cancer. Oncogenic mutations in the gene encoding the p110α catalytic subunit, *PIK3CA*, are common in breast, colon, and endometrial cancers [28,29,30,31,32]. Somatic, missense mutations have been identified throughout the sequence of p110α. Interestingly, about 80% of these mutations are concentrated at 3 ‘hotspots’, Glu542Lys, Glu545Lys and His1047Arg [28,29]. The PI3Kα selective inhibitor, alpelisib (NVP-BYL719), recently completed Phase III clinical trials with encouraging results in patients with tumors harboring *PIK3CA* mutations (Table 1) [33]. 

Mutations are not common in the other isoforms, but overexpression has been linked to cancer [8,34,35]. Cancers deficient in the phosphatase PTEN have been linked with increased PI3Kβ expression and activity. Studies in PTEN-deficient cell lines, as well as conditional knock-out mice have shown that PI3Kβ inactivation blocks prostate cancer development [36,37,38,39,40]. A number of PI3Kβ selective inhibitors are currently in Phase I/II trials for PTEN-deficient cancers, including AZD8186 and GSK2636771 (Table 1) [41]. Rapid clearance of SAR260301 halted its clinical development [42].

Although the roles for PI3Kα and PI3Kβ were the first to be characterized, the first PI3K inhibitor to be approved for use was the PI3Kδ selective inhibitor, idelalisib. Constitutively active PI3Kδ signaling has been shown in many B-cell malignancies [43,44,45]. Idelalisib is a Food and Drug Administration (FDA) approved as a combination therapy with rituximab in relapsed chronic lymphocytic lymphoma (CLL) and as a monotherapy in relapsed follicular lymphoma (FL) and small cell lymphocytic lymphoma (SLL) (Table 1) [46,47,48]. Although effective in treating lymphoma, unpredictable immune-mediated toxicity is limiting its use for inflammatory diseases and non-cancer indications [49,50]. Two other PI3K inhibitors have also recently been approved: duvelisib (PI3Kγ/δ) for relapsed CLL or SLL [51,52] and copanlisib (pan-PI3K) for relapsed FL [53,54,55,56]. 

PI3Kγ has been implicated with a key role in the tumor microenvironment [57]. PI3Kγ signaling is activated in myeloid cells in response to tissue hypoxia [58,59,60,61]. PI3Kγ deletion and kinase-dead knock-in studies in mice have also demonstrated a vital role for PI3Kγ in the myeloid cells that make up the immune-suppressive tumor microenvironment [60,61,62,63]. The PI3Kγ selective inhibitor, IPI-549 is undergoing clinical evaluation in patients with advanced solid tumors (Table 1) [57].

The development of isoform selective inhibitors has been vital to uncovering the unique functions of each isoform and their corresponding therapeutic potential. Significant progress has been made, and isoform selective inhibitors are now available for each of the four Class I isoforms. They continue to be useful in uncovering important details of PI3K physiology and the understanding of cancer signaling. While the clinic will ultimately decide about the efficacy and the relative benefit of pan and isoform selective inhibition, an understanding of the underlying mechanisms of isoform selectivity will continue to hold importance as resistance develops and the next generation of inhibitors is required. In this review, we will first focus on the available structural information for each of the isoforms, and then how this informs isoform selective inhibitor design and development. Pan PI3K inhibitors have been reviewed in detail elsewhere and will not be addressed in this review [64].

## 2. Structural Biology of PI3K

The foundation for structure-based drug design is the availability of medium to high-resolution structures. p110γ, the sole-member of Class IB, was the first PI3K to be crystallized [77]. Stable expression of p110γ in insect cells was achieved with an N-terminal truncation of the Adaptor Binding Domain (ABD), which mediates the interaction between p110 and the regulatory subunit [77]. This construct is hereafter referred to as ΔABD. 

**Figure 1:**
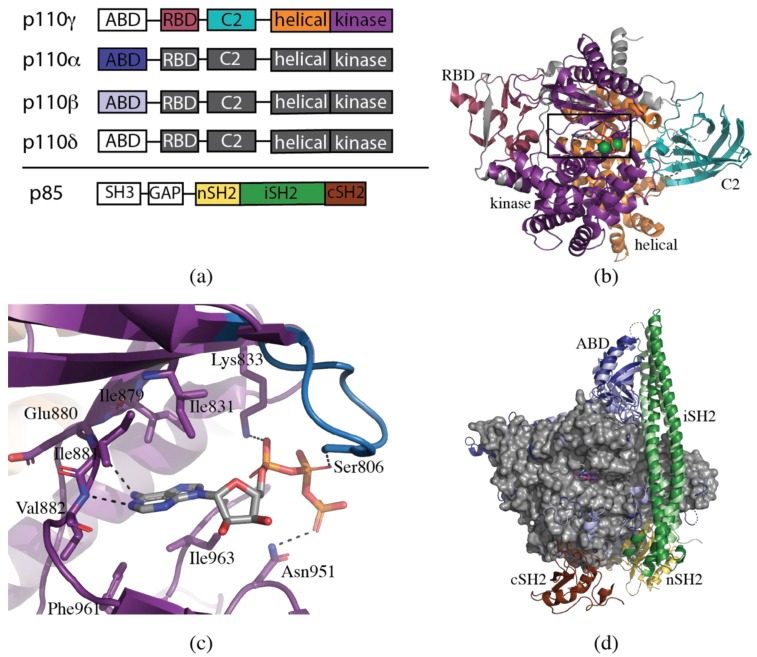
Structural biology of PI3K. (**a**) Domain organization of the catalytic subunits (p110) and regulatory subunit (p85) of Class I PI3K. Domains colored in white have not been crystallized in PI3K complex structures. (**b**) The structure of ΔABD-p110γ in complex with ATP (PDB ID 1E8X). The domains are colored according to (**a**). Lutetium ions are shown as green ions, approximating the position of magnesium. The ATP binding site is within the black rectangle. (**c**) ATP binding site (PDB ID 1E8X). The adenine ring forms hydrogen bonds with the backbone of γGlu880 and γVal882 and stacks between γIle831 and γIle963. The α-phosphate interacts with γLys833, β-phosphate with γSer806 and γ-phosphate with γAsn951. The P-loop has been highlighted in blue. (**d**) The structure of Class IA PI3K. p110δ is shown in gray as surface representation (PDB ID 2WXL). The ABD of p110α (dark blue, PDB ID 4OVU) and p110β (light blue, PDB ID 2Y3A) are shown in cartoon representation, mediating the binding to iSH2 of p85 (α, dark green; β in light green). The nSH2 from PI3Kα is shown in yellow, and the cSH2 from PI3Kβ is shown in brown.

All the Class I p110 isoforms consist of five domains (Figure 1a). The structure of ΔABD-p110γ revealed the overall architecture of four of these domains, which were subsequently shown to be highly homologous to the other isoforms (PDB ID 1E8X, Figure 1b). The Ras-binding domain (RBD) in p110γ (residues γ220-311) contains a five-stranded β-sheet flanked by two α-helices, a similar fold to the RBD of two other well-known Ras effectors, Raf and RalGDS [78,79]. The RBD is situated in close proximity to the kinase domain, suggesting that Ras may activate p110γ via an allosteric mechanism. The fold of the C2 domain of p110γ (residues γ357-522) is analogous to that of PLCδ1, containing an eight-stranded antiparallel β-sandwich [80]. The C2 domain is postulated to be involved in membrane binding. The helical domain (residues γ545-725) consists of five pairs of antiparallel α-helices. The precise function of this domain is unknown. The fold of the kinase or catalytic domain (residues γ726-1092) has a high level of similarity to that generally observed in protein kinases. It is a two-lobed structure, with ATP binding at the hinge region between these two lobes [77].

These initial structures of ΔABD-p110γ revealed a number of important features of the ATP-binding site, which is of particular interest to the development of inhibitors (Figure 1c). In a similar fashion to that observed in protein kinases, N1 and N6 of the adenine ring system form complementary hydrogen bonding interactions with the amide backbone of γGlu880 and γVal882 at the hinge region between the N- and C-lobes of the kinase domain. The adenine ring moiety is sandwiched between hydrophobic residues (γIle831, γIle879, γPhe961 and γIle963) at the base and roof of the binding site. The ribose ring projects towards hydrophobic region II without making any specific interactions with the protein. The phosphate tail of ATP interacts with a loop designated the P-loop (p110γ residues 803–811), analogous to the glycine-rich loop in protein kinases. The α-phosphate interacts with γLys833, the β-phosphate with γSer806 and the γ-phosphate with γAsn951 [81].

It was another seven years before the structure of a Class IA PI3K was published [82]. One of the major complicating factors in the crystallization of the Class IA PI3K isoforms is the requirement to co-express with p85 for stability. Full-length p85 has an SH3 domain, Bcl-2 homology (BH) domain and two SH2 domains, connected by the inter-SH2 (iSH2) domain (Figure 1a). The minimum construct of p85α required for stable expression of p110α is the N-terminal SH2 (nSH2) and the iSH2 domains [83]. In 2007, Huang et al. [82] published the wild-type structure of p110α in complex with this truncated construct of p85α (PDB ID 2RD0) (Figure 1d). Despite only 35% sequence identity, the overall fold of p110α displays a high degree of similarity to p110γ. The iSH2 domain is an extended coiled-coil which interacts with p110α via the ABD and C2 domains. The electron density for the nSH2 domain of p85α was disordered, and the domain was not able to be traced [82]. Two years later, the structure of an oncogenic mutant PI3Kα, H1047R, was determined in the presence of wortmannin (PDB ID 3HHM) [84]. The slightly higher resolution allowed for the tracing of the nSH2 domain.

Crystal structures of murine p110δ followed in early 2010 [85]. A tobacco etch virus (TEV) protease cleavage site was introduced between the ABD and the RBD. A complex containing p110δ and iSH2 of p85α was expressed, and then the ABD cleaved with TEV protease, leaving just the N-terminally truncated ΔABD-p110δ (Figure 1d). A total of 14 structures were determined, including the holo-enzyme and the enzyme in complex with a range of pan-PI3K and PI3Kδ selective inhibitors [85].

The last isoform to be crystallized was PI3Kβ [86]. Unlike p110α, it was found that the C-terminal SH2 domain (cSH2) of p85 was more important than the nSH2 for stabilization and inhibition of p110β [86]. For crystallization, therefore, murine p110β was expressed in complex with the iSH2 and cSH2 domains of p85β. No structure of the holo-enzyme was reported, only that in complex with the pan-PI3K inhibitor, GDC-0941 (PDB ID 2Y3A) (Figure 1d). It should be noted that the human p110β has a N-terminal extension of six amino acids compared with the mouse, which affects the numbering of amino acids. The numbering for human p110β, which differs from the crystal structure numbering, will be used in this paper. 

Inhibitor bound structures have continued to be published for each of the isoforms. For PI3Kγ, a total of 97 structures have currently been deposited to the Protein Data Bank (PDB) (as of 25 January 2019) [87]. The first crystal structures of PI3Kα, while revealing key insights into mechanisms of regulation, were of limited use for structure-based drug design, as the soaking of substrate or inhibitors was precluded by the binding of the RBD of a symmetry-related molecule in the ATP-binding site. Hon et al. [88] were able to co-crystallize an inhibitor with a similar construct using different crystallization conditions (PDB ID 4A55). However, a resolution of 3.5 Å is still less than ideal for revealing molecular details of binding and insights into inhibitor selectivity. In an attempt to address these issues, three further constructs have been published that yield higher resolution, inhibitor bound structures. Firstly, a double mutant in the RBD (M232K, L233K) was engineered to influence crystal packing. The mutant packs differently, such that the linker between the ABD and RBD from a neighboring molecule inserts in the space near the ATP-binding site, but does not occlude it, allowing for the soaking or co-crystallization with inhibitors [65,89]. The two crystal structures of this construct in the PDB (PDB ID 4JPS, 4ZOP) are higher resolution (2.2 Å and 2.6 Å) than the wild-type structure (2.9–3.5 Å). Another alternative was an N-terminal fusion of p85-niSH2 to p110α, with a glycine/serine linker between the two (PDB ID 4L1B, 4L23, 4L2Y, 4YKN) [90,91]. This has the benefit of increasing homogeneity of the complex, and results in a corresponding increased resolution compared with the original wild-type structures (2.5–2.9 Å). Finally, an isolated p110α ΔABD construct with a C-terminal truncation of lipid binding residues, was also produced (PDB ID 4TUU, 4TV3) [92]. While not catalytically active, this construct still binds PI3Kα inhibitors and crystallizes with medium resolution (2.6–2.8 Å). A total of 41 structures of PI3Kα have been deposited in the PDB (as of 25 January 2019). Representative structures from each of the constructs overlay well with the original wild-type structure (PDB ID 2RD0), with RMSDs ranging from 0.7–1.1 Å. 

Only one further murine PI3Kβ crystal structure has been published, using a similar ΔABD construct to that of ΔABD-p110δ [71]. A total of 46 PI3Kδ structures have been deposited into the PDB (as of 25 January 2019). In addition to the murine ΔABD-p110δ structure, human p110δ/iSH2 structures have also been published (PDB ID 5T8F, 5UBT, 5VLR), with lower resolution than the ΔABD structures (2.8–2.9 Å) [93,94,95].

## 3. Structural Determinants of Isoform Selectivity

Class I PI3Ks have a highly homologous ATP binding site that only differs in a handful of residues at the periphery of the binding site. These non-conserved residues cluster into two regions, designated Regions 1 and 2, which influence inhibitor selectivity [96,97]. The growing collection of structure-selectivity-activity relationships (SSAR) from extensive medicinal chemistry efforts has revealed that understanding the structural basis of isoform selectivity is far from a simple task. It is the complex, combinatorial product of differences in non-conserved residues, conformational flexibility, hydrogen bond networks, and interactions in the varying regions of the binding site. We have collated the available data gained from X-ray crystallography, computational modeling, and mutagenesis studies to provide an analysis of the various mechanisms of selectivity. We will first present a road-map of the various regions of the ATP-binding site, and then discuss the influence of these regions on the selectivity of various isoform selective inhibitors. The extensive literature on PI3K inhibitors, which has been the subject of a recent review (418 patents and 192 medicinal chemistry publications since 2012 [64]), precludes a detailed analysis of every inhibitor. In this review, we have chosen to concentrate on inhibitors with published data on isoform selectivity as well as structural information on binding mode, and particularly those inhibitors that yield insight into general principles of the structural determinants of isoform selectivity. 

### 3.1. Regions of the Binding Site

#### 3.1.1. Hinge-Region

**Figure 2:**
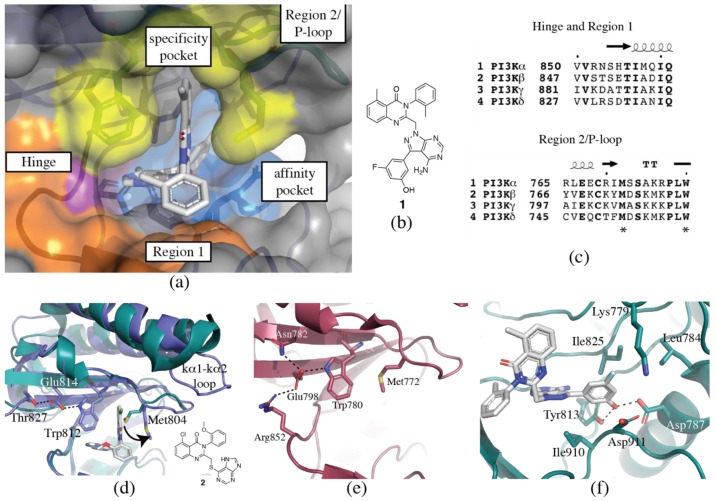
Regions of the binding site. (**a**) Overall picture of the inhibitor binding site with **1** bound (p110δ, PDB ID 2WXG). The hinge region is shown in purple, the affinity pocket in blue and the open specificity pocket in yellow. Region 1 is shown in orange. The P-loop is shown in teal. (**b**) Structure of **1**. (**c**) Sequence alignment of hinge and Regions 1 and 2. (**d**) The specificity pocket in p110δ and p110γ. p110δ is shown in teal (PDB ID 2WXG) and p110γ is shown in slate (PDB ID 2CHW). The movement of γMet804 required for opening of the specificity pocket is marked by an arrow. PIK39 **2** is shown in the binding site. The extended loop between the kα1 and kα2 helices in p110γ is indicated, along with the stabilizing hydrogen bond interactions with γTrp812. (**e**) Stabilizing interactions of αTrp780 in p110α (PDB ID 4JPS). (**f**) The affinity pocket. The structure of **1** bound to p110δ (PDB ID 2WXG), with key residues in the affinity pocket marked.

One of the most important features of kinase inhibitors is the presence of a “hinge-binder” (Figure 2a,b). The ATP binding site sits at the interface between the two lobes of the kinase domain, and the presence of a hinge-binding motif, such as a morpholinyl or purinyl substituent, mimics the adenine ring of ATP and acts to anchor the inhibitor in the binding site via hydrogen bonds to the backbone of γIle881 (valine in α, β and δ) and/or γVal882 (conserved across all isoforms). The presence of this hinge interaction is preserved in all inhibitor-bound structures determined to date. Although these two residues are invariant or highly similar between the isoforms (valine-isoleucine), the adjacent residues (γLys883, γAsp884 and equivalent) are not conserved, which may present opportunities for modifications of the hinge-binding moiety to influence selectivity (Figure 2c). 

#### 3.1.2. Specificity Pocket

The specificity pocket was first identified in the PIK39-p110γ crystal structure (PDB ID 2CHW, Figure 2d) [9]. PIK39 (**2**) is a PI3Kδ selective inhibitor that exhibits mid-nanomolar potency at PI3Kδ, and 100x selectivity over PI3Kβ and PI3Kγ, and no inhibition of PI3Kα at concentrations up to 100 µM [9]. The inhibitor induces an outward movement of a conserved methionine residue on the P-loop to reveal a hydrophobic binding pocket, designated the specificity pocket. Inhibitors of this type are said to bind in a “propeller” conformation, as opposed to the relatively flat conformation of inhibitors that do not access the specificity pocket. As it was originally discovered in PI3Kδ selective inhibitors, opening of the specificity pocket was thought to confer PI3Kδ specificity, but now PI3Kβ and PI3Kγ selective propeller-shaped inhibitors have also been discovered, suggesting the situation is more complex [57,71,98]. The residues directly involved in the opening of the specificity pocket are conserved between the isoforms, but there is computational and biochemical evidence to suggest that non-conserved residues surrounding the pocket may variously influence the accessibility of the pocket. Such evidence suggests that the specificity pocket may be most energetically accessible in PI3Kβ and δ. However, the existence of PI3Kγ and PI3Kγ/δ selective propeller-shaped inhibitors indicates a level of accessibility in PI3Kγ, too; this may be due to other features of the inhibitor which overcome the energetic restrictions to the pocket opening, or it may be more easily accessed than initially anticipated.

A comparison of **2** in both p110γ and p110δ (PDB ID 2WXF) crystal structures revealed that the movement in p110δ is limited to local changes in the P-loop, whereas opening of the specificity pocket in p110γ requires a more extensive conformational change that propagates through the P-loop into the helices of the N-lobe [9,85]. Sequence differences between the isoforms may account for variations in the conformational plasticity of the P-loop. p110γ has an extended loop between the kα1 and kα2 helices, which is absent in the three Class IA isoforms (Figure 2d). This loop packs next to the P-loop and may contribute to differences in flexibility [85]. 

Molecular dynamics simulations showed that in p110δ opening of the pocket involves a synchronized movement between δTrp760 and δMet752 [85]. In p110γ, however, γTrp812 (equivalent to δTrp760) is constrained by a hydrogen bond to γGlu814, which in turn interacts with γThr827. This hydrogen bond network reduces the flexibility of γTrp812, thus potentially disfavoring the opening of the specificity pocket (Figure 2d) [85]. 

The specificity pocket has now been observed in crystal structures of all Class I isoforms. A comparison between the ‘closed’ (PDB ID 4JPS) and ‘open’ (PDB ID 4A55) p110α crystal structures reveals a similar network of hydrogen bonds restricting tryptophan movement to that found in p110γ: αTrp780 (equivalent to δTrp760) is restrained by a hydrogen bond to αGlu798, which in turn is held in place by interactions with αArg852 and αAsn782 (Figure 2e). In the ‘open’ conformation, the hydrogen bond to αGlu798 is maintained, however the anchoring interactions with αArg852 and αAsn782 are absent. It should be noted that the resolution of the ‘open’ structure is only 3.5 Å, and detailed information on the positioning of side-chains may not be present [88]. Analysis of the available SSAR data surrounding the specificity pocket reveals that the propeller-shaped inhibitors consistently show the highest selectivity over PI3Kα [85]. The hydrogen bond network restricting αTrp780 movement may contribute to this, but it is also possible that there are more complex interactions restricting P-loop flexibility in p110α. 

A detailed comparison between the ‘closed’ (PDB ID 2Y3A) and ‘open’ (PDB ID 4BFR) p110β structures is confounded because of the different constructs used for crystallization [71,86]. The ‘closed’ structure has the iSH2 and cSH2 domains from p85β, while the ‘open’ structure is ΔABD. There are some significant conformational changes between the two structures, however these are more likely to be caused by the presence or absence of the p85β domains. Neither of the structures reveals a constricting hydrogen bond network to βTrp781, presumably contributing to the relative ease of opening of the specificity pocket in PI3Kβ compared with PI3Kα.

A reciprocal mutagenesis study identified a non-conserved residue in PI3Kβ, βTyr778, adjacent to the conserved methionine that may also contribute to the extent of conformational plasticity of the P-loop [98]. The residue in this position is aliphatic in p110α and p110γ (isoleucine and valine, respectively), but aromatic in p110β and p110δ (tyrosine and phenylalanine, respectively), which may account for the similar profiles of inhibitors for these two pairs of isoforms. 

Each of these mechanisms likely contribute to the apparent differences in accessibility of the specificity pocket between the four isoforms. However, it is also apparent that inhibitor selectivity is not simply determined by the opening of the specificity pocket, but rather a complex amalgamation of inhibitor interactions throughout the binding site. 

#### 3.1.3. Affinity Pocket

The affinity pocket is a pocket that is not accessed by ATP, and is surrounded by the conserved residues δLys779, δAsp787, δLeu791, δTyr813, δIle825 and δAsp911 (Figure 2f) [85]. Extension of inhibitors into this pocket has been shown to increase potency and despite consisting of only invariant residues, can also surprisingly modulate selectivity. Differences in selectivity may be influenced by distinct hydrogen bonding networks to non-conserved residues surrounding the pocket. This will be discussed more below with specific inhibitor examples. 

#### 3.1.4. Non-Conserved Regions

We have previously described two regions of non-conserved residues that contribute to isoform selectivity [96,97]. Region 1 (alternatively described as hydrophobic region II or ribose binding pocket), which is a region of eight amino acids C-terminal to the hinge region (δ829-836), has four positions of complete variability between the four isoforms, and two positions with a single isoform not conforming (Figure 2a,c). Isoform specific interactions with this region have been shown to be vital in the development of isoform selective inhibitors. Region 2 coincides with the P-loop, and while less variable than Region 1, key amino acid differences in this region still influence selectivity via specific interactions and may also affect the conformational plasticity of this loop and accessibility of the specificity pocket (Figure 2a,c). 

### 3.2. PI3Kδ Selective Inhibitors

**Figure 3:**
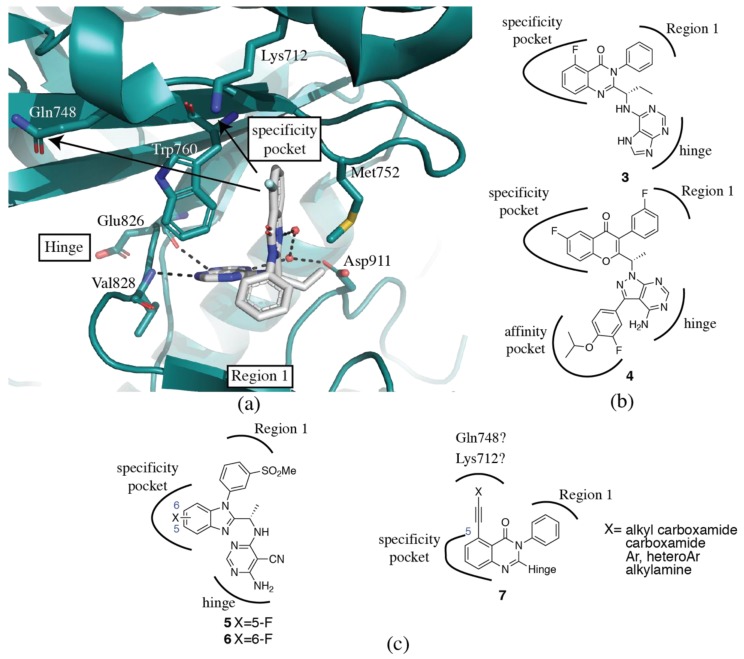
Modifications of the specificity pocket binding moiety. (**a**) Idelalisib (**3**) bound to p110δ (PDB ID 4EX0). Substituents at the 5-position of **7** are predicted to interact with either δLys712 or δGln748. (**b**) Structure of idelalisib (**3**) and umbralisib (**4**). (**c**) Structures of **5**–**7**.

The clinically approved PI3Kδ inhibitor, idelalisib (**3**) along with the clinical candidate umbralisib (**4**) are both propeller shaped PI3Kδ selective inhibitors (Table 1). Idelalisib (**3**) (PDB ID 4XE0) binds to p110δ in a similar fashion to PIK39 (**2**) (Figure 3a,b) [74]. The purine binds to the hinge region, with N3 and N9 forming hydrogen bonds with the backbone carbonyl and nitrogen of δGlu826 and δVal828, respectively. The N7 of the purine forms a water-mediated hydrogen bond network with δAsp911 and N1 of the quinazolinone. The substituted quinazolinone moiety binds in the specificity pocket, with the phenyl group orienting perpendicular to the quinazolinone and extending toward Region 1. The structure of umbralisib (**4**, Figure 3b) is related, with a 4-amino-pyrazolo-pyrimidine serving as the hinge binder and a chromone binding in the specificity pocket. It has traditionally been accepted that binding in the specificity pocket confers PI3Kδ selectivity, but as has been discussed above, the emerging picture is significantly more complicated. So what sets PI3Kδ selective propeller inhibitors apart from PI3Kβ, PI3Kγ and dual selectivity inhibitors? The impact of the hinge region, affinity pocket and Region 1 (targeted by the ‘third blade’ of the propeller) cannot be underestimated and will be discussed in more detail below. As for the specificity pocket moiety, the most common motifs in PI3Kδ selective inhibitors are 6,6 and 5,6-fused heterocyclic aromatic ring systems, which have been reviewed in detail by Perry et al. [49]. Halogen substitutions at the 5- and 8-positions of a quinazolinone core show a 10x range in PI3Kδ potency and some limited variation in selectivity (Table 2) [99]. Such substitutions are predicted to modify the basicity of N1, which in the related crystal structure of p110δ-idelalisib (**3**) makes a water-mediated hydrogen bond with δAsp911. The 5,8-dichloro analog **10** has a dramatic increase of selectivity over PI3Kγ (470x) compared with the monochloro analogs, **8** and **9** (120x and 183x, respectively). The N1 pK_a_ is reduced from 1.97 (**8**) and 1.72 (**9**) in the monochloro analogs to 0.48 (**10**), at first suggesting a correlation; however, the pK_a_ in the 8-chloro-5-fluoro analog **11** is further reduced to 0.28, and the δ/γ selectivity is reduced back to 127x. A clear structural rationale is unavailable to account for these dramatic selectivity differences from subtle chemical changes. In another series of quinazolinone inhibitors, the 6-fluoro substituent in **12** dramatically reduces selectivity over PI3Kα to only 8x [100]. Combining this with an 8-chloro substitution in **13** restores selectivity to >100x. In a similar series reported by Patel et al. [101] (Table 2), a 6-F substitution (**15**) has a limited effect on δ/α selectivity. 5-halogen substituents (**16**) show improvement in PI3Kδ potency and selectivity compared with an unsubstituted ring. Other series expounding SSAR on limited halogen substitution around quinoline and quinoxaline cores have also been published [72,102]. In a series of benzimidazole based inhibitors, 5- or 6-fluoro substituents made significant differences in selectivity, from >1800x (**6**) to 189x (**5**) at PI3Kα, and less so over PI3Kβ (52x (**6**), 181x (**5**)) and γ (42x (**6**), 79x (**5**)) (Figure 3c) [103]. Longer or bulkier substitutions at the 5-position of a quinazolinone have also been explored (**7**) (Figure 3c) [104]. Although no crystal structures of these compounds bound to PI3K are available, docking studies suggest that these substitutions may access non-conserved δGln748 (α: Glu, β: Lys, γ:Lys) and δLys712 (α:Met; β: Lys; γ:Ile), which may contribute to their selectivity (Figure 3a) [104]. It is clear from the limited SSAR available that substitutions around the bicyclic specificity pocket core can modulate both PI3Kδ potency and selectivity, although a structural rationale for many of these differences is elusive. Specific features of PI3Kβ and γ propeller-shaped inhibitors will be discussed further in the following sections.

**Figure 4:**
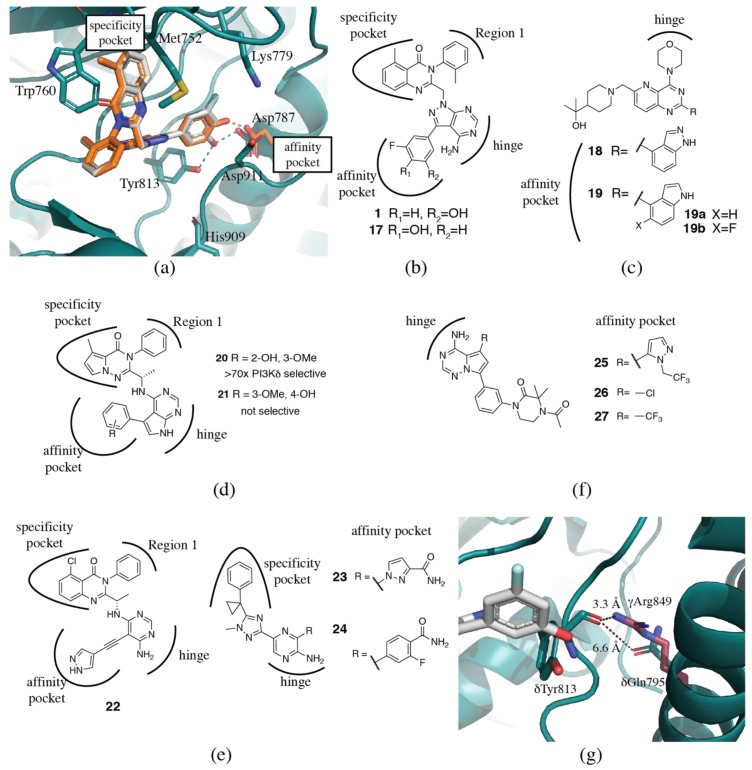
Modifications in the affinity pocket. (**a**) Impact of subtle differences in the affinity pocket. *meta*-substituted fluorophenol **1** (white, PDB ID 2WXG) can form hydrogen bonds with δAsp787 and δTyr813 (indicated with teal dashed lines), while *ortho*-substituted fluorophenol **17** (orange, PDB ID 2WXH) can only form hydrogen bonds with δAsp787 (indicated with orange dashed lines) (**b**) Structures of **1** and **17** (**c**) Structures of inhibitors with indole and indazole affinity pocket binding motifs (**d**) Structures of inhibitors **20**–**21** (**e**) Structures of inhibitors **22**–**24** (**f**) Structures of inhibitors **25**–**27** (**g**) Rationalizing differences in affinity pocket selectivity. γArg849 forms a hydrogen bond with the backbone of γTyr867 (equivalent to δTyr813), restricting movement in the affinity pocket, while the side-chain of δGln795 is too short to make the same interaction. Distances are marked with dashed lines. The fluorophenol moiety of **1** bound in the affinity pocket is shown for reference.

It is well established that extension of the inhibitor into the affinity pocket not only increases the potency of inhibitors but can also modulate selectivity. In the SW series of inhibitors [85,105], a *meta*-fluorophenol binding moiety in the affinity pocket yields a potent PI3Kδ inhibitor (**1**) with >300x selectivity against PI3Kα and PI3Kβ, and almost 50x selectivity over PI3Kγ (PDB ID 2WXG). A simple change to an *ortho*-substituted fluorophenol (**17**) reduces potency at PI3Kα, β and δ, while increasing potency at PI3Kγ (PDB ID 2WXH). The result is an inhibitor that maintains good selectivity over PI3Kα and β (1000x and 77x, respectively), but completely loses selectivity over PI3Kγ [85]. The hydroxyl group in **1** can hydrogen bond to both the conserved δAsp787 and δTyr813, while **17** can only form a hydrogen bond to δAsp787 (Figure 4a,b). This may explain the loss of potency at PI3Kα, β and δ, but does not explain why the inhibitor gains potency at PI3Kγ. The affinity pocket is comprised of conserved residues, making these selectivity differences difficult to rationalize. 

The impact of subtle differences in the affinity pocket binding moieties has been also been observed by others. In a series of non-propeller shaped PI3Kδ selective inhibitors, the change of an indazole (**18**) for an indole moiety (**19**) in the affinity pocket dramatically reduces the δ/α selectivity of the inhibitors (Figure 4c) [106]. The indole has 200-600x selectivity for PI3Kδ over the other three isoforms. When replaced with an indazole, the potency for PI3Kα improves by 7.5x, while equivalent potency is maintained at PI3Kβ and γ. Potency at PI3Kδ shows a modest 2x decrease. The authors propose alternate hydrogen bonding networks as influencing the selectivity. In the crystal structure (PDB ID 4EZJ), the indole nitrogen in **19b** (a fluorinated analog of **19** that has similar potency and selectivity) forms a hydrogen bond with γAsp841 (δAsp787), while the CH is positioned 3.1 Å from the γTyr867 (δTyr813) hydroxyl, which in turn hydrogen bonds to the backbone carbonyl from γHis962 (δHis909). The indazole **18** (PDB ID 4EZK), however, can hydrogen bond to both γAsp841 and γTyr867, which forces a break in the hydrogen bond between γTyr867 and γHis962. The authors propose that this change may be more easily accommodated in the p110δ isoform [106]. The rationale for the PI3Kα potency increase is unclear. Erra et al. [107] also found that modulations in the affinity pocket could influence selectivity over PI3Kα. Changes to the phenyl substitution pattern greatly affected selectivity (**20**, **21**, Figure 4d). The authors hypothesize that a double interaction with both δLys779 and δAsp787 could compensate for the energy cost needed to open the specificity pocket in PI3Kα [107]. However, in a series of pyrrolotriazinone propeller shaped compounds, a pyrazole moiety in the affinity pocket (**22**) forms a triple interaction with the conserved δAsp787, δLys779 and δAsp911 (PDB ID 5I6U) and maintains good selectivity over PI3Kα (Figure 4e) [101]. 

In a series of triazole aminopyrazine PI3Kδ inhibitors, modulation of the affinity pocket binding moiety was able to increase PI3Kδ potency and selectivity against the other isoforms from 63 nM with 100-500x selectivity (**23,** PDB ID 5T2L) to 0.8 nM with 2000-25000x selectivity (**24**, PDB ID of des-fluoro analog, 5T2M) (Figure 4e). Interactions with δTyr813 and δAsp911 and water interaction networks seem to be key to PI3Kδ potency and selectivity over the other isoforms [108]. Substitutions at the 5-position of a series of pyrrolotriazines that extend into the affinity pocket were able to increase selectivity against the PI3Kγ isoform [93]. Replacement of a 1-(2,2,2-trifluoroethyl)-1-pyrazole (**25**, PDB ID 5VLR) with a chloro substitution (**26**) increased selectivity over PI3Kγ from 37x to 180x. Replacement of the chloro with a trifluoromethyl group (**27**) showed similar selectivity over PI3Kγ (Figure 4f) [93]. 

One potential explanation for the apparent higher tolerance of PI3Kδ for substitutions in the affinity pocket is a non-conserved residue beyond the affinity pocket. In p110γ crystal structures, γArg849 forms a hydrogen bond with the backbone of γTyr867, presumably restricting the flexibility of this residue (Figure 4g). The residue at the equivalent position in p110α and β is also an arginine. However, in p110δ, the residue at the equivalent position is δGln795, and the shorter sidechain length precludes formation of a hydrogen bond and may allow for greater flexibility. 

**Figure 5:**
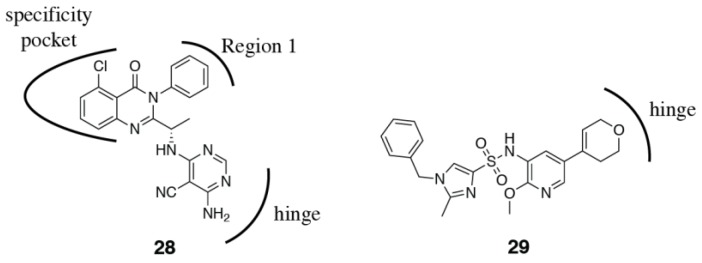
Structures of **28** and **29**, which demonstrate differences in hinge-binding moieties that affect isoform selectivity.

Surprisingly, even modification of the hinge binding moiety can influence selectivity. Like the affinity pocket, the hinge residues are conserved between the four isoforms. Modification of the hinge-binder of duvelisib from the purine ring to the 4-amino,5-cyano-pyrimidine **28** was able to increase selectivity for PI3Kδ from 55-1200x to 200-4600x (Figure 5) [101]. A recent paper from GSK have investigated the combination of a pyridine sulfonamide, which they describe as a privileged PI3Kδ fragment, with a series of hinge binding moieties (**29**, Figure 5) [109]. They observe marked differences in isoform selectivity that is dependent on the hinge binding moiety. It is very difficult to rationalize these differences in selectivity.

**Figure 6:**
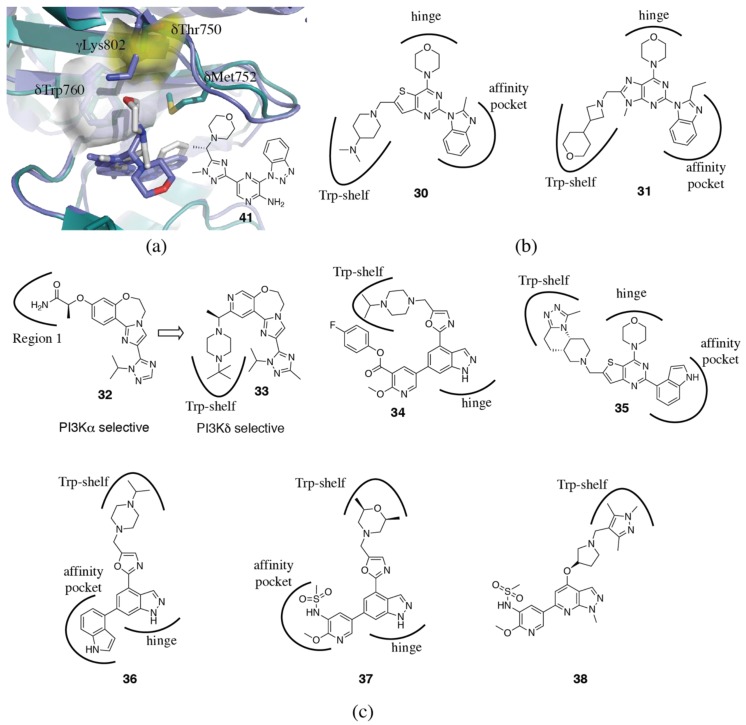
The tryptophan shelf. (**a**) The tryptophan shelf is formed by the exposed surface of δTrp760 (p110δ cartoon shown in teal, the surface of δTrp760 shown in white, PDB ID 5T27) by the small side-chain of δThr750 (surface shown in yellow). In p110γ (slate, PDB ID 5T23), the longer side-chain of γLys802 occludes the surface of the tryptophan. The different conformations of **41** bound to p110δ (inhibitor colored in white) and p110γ (inhibitor colored in purple) are shown. (**b**) Structures of **30**–**31**. (**c**) Structures of **32**–**38**.

In addition to the PI3Kδ selective inhibitors that access the specificity pocket, there are a number of other inhibitors which utilize the so-called “tryptophan shelf” that is present in PI3Kδ [106]. The tryptophan shelf is formed by the non-conserved δThr750, which allows inhibitors to access the face of the conserved δTrp760, which forms the tryptophan shelf. In p110α, β and γ, this residue is either an arginine (αArg770) or a lysine (βLys777 and γLys802), which occludes the face of the tryptophan from access by inhibitors (Figure 6a). Sutherlin et al. [106] describe a new class of pyrido-pyrimidine PI3Kδ selective inhibitors (**19a** and **b**) that show greater than 200x selectivity without accessing the specificity pocket (PDB ID 4EZJ) (Figure 4c). Interestingly, mutagenesis of γLys802 to a threonine to mimic the tryptophan shelf in PI3Kδ did not significantly alter the potency of the inhibitors for PI3Kγ. The authors suggest, in a similar fashion to that discussed for the specificity pocket, that further mutations around γTrp812 may be needed to recapitulate the flexibility of this residue in p110δ, and thus the affinity of inhibitors for this isoform [106]. In another series of Genentech tryptophan shelf inhibitors, modification of the core influences the dihedral angle of the tryptophan shelf binding moiety and optimization of the interaction with δTrp760 improves PI3Kδ selectivity (**30, 31**, Figure 6b) [110,111]. Bulkier substituents further improved PI3Kδ selectivity, as they are less able to be accommodated by the bulkier arginine and lysine residues in PI3Kα, β and δ [111]. Safina et al. [112] describe the rational design of a PI3Kδ inhibitor (**33**) from a PI3Kα selective inhibitor (GDC-0326, **32**) by introducing a tryptophan shelf substituent and optimizing the dihedral angles to position it for interaction with δTrp760 (Figure 6c). Dalton et al. [113] describe the development of a covalent PI3Kδ inhibitor (**34**) that achieves its isoform selectivity via the tryptophan shelf, and covalently interacts with the conserved lysine, δLys779 (PDB ID 6EYZ) (Figure 6c). Schwehm et al. [114] have reported the design of a novel tricyclic scaffold (**35**) which can be used as a tryptophan shelf binding motif (Figure 6c). Clinical candidates GSK2269557 (**36**) and GSK2292767 (**37**) both use the tryptophan shelf (PDB ID 5AE8, 5AE9) (Figure 6c) [73], as do a series of pyrazolopyridines described by Hamajima et al. (**38**, Figure 6c) [115].

**Figure 7:**
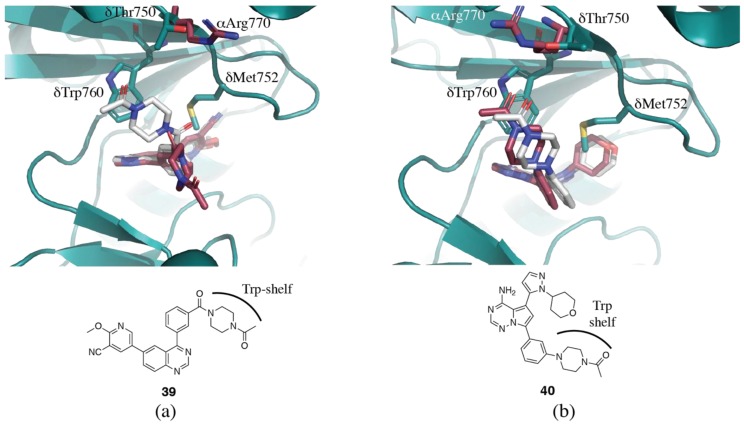
Comparing tryptophan shelf inhibitors in p110α and p110δ. (**a**) **39** bound to p110δ (**39** in white, p110δ in teal, PDB ID 5IS5) and p110α (**39** in raspberry, PDB ID 5ITD). For simplicity, the cartoon structure of p110α has not been shown. The conformation of αArg770, which restricts access to the tryptophan shelf in PI3Kα, has been shown. (**b**) **40** bound to p110δ (**40** in white, p110δ in teal, PDB ID 5UBT) and p110α (**40** in raspberry, PDB ID 5UBR). The conformation of αArg770, which restricts access to the tryptophan shelf in PI3Kα, has been shown, while the cartoon structure of p110α has been removed for clarity.

To give further insight into how these families of inhibitors achieve their selectivity, crystal structures of the same inhibitor have been determined in two isoforms [94,108,116]. The crystal structure of the quinazoline-based inhibitor **39** in p110δ (PDB ID 5IS5) reveals that the acetyl-piperazine group of the ligand stacks with δTrp760 using the tryptophan shelf (Figure 7a) [116]. In p110α (PDB ID 5ITD), however, the inhibitor cannot bind in the same conformation due to the presence of αArg770 π-stacking with αTrp780 (equivalent to δTrp760). As a result, the inhibitor binds in an extended conformation in p110α and loses the hydrophobic interactions present in the p110δ complex, resulting in the inhibitor’s preference for p110δ [116]. A follow up paper describes further development of this series of inhibitors to optimize the conformation and hydrophobic interactions with δTrp760 [75]. Conversely, a similar set of p110α (PDB ID 5UBR) and p110δ (PDB ID 5UBT) structures show that the pyrrolo-triazine inhibitor **40**, binds to p110α and p110δ in the same conformation (Figure 7b) [94,117]. In p110δ, the acetylpiperazinyl moiety sits at the tryptophan shelf, and the acyl group forms a hydrogen bond with the hydroxyl from δThr750. In p110α, αArg770, which normally occludes the tryptophan shelf, has shifted out of the way to allow the inhibitor to stack with the tryptophan. However, there are no residues for the acyl group to hydrogen bond to. The requirement for the shift of the arginine and the lack of hydrogen bond probably contribute to the lower potency observed for PI3Kα. Further optimization of these inhibitors has been published [118]. A series of triazolo aminopyrazines also utilize the tryptophan shelf for PI3Kδ selectivity. Crystal structures of **41** in both p110γ (PDB ID 5T23) and p110δ (PDB ID 5T27) show that the morpholinyl substituent extends toward the tryptophan shelf in PI3Kδ, but must rotate in p110γ, since access to the tryptophan residue is blocked by γLys802(Figure 6a) [108]. 

**Figure 8:**
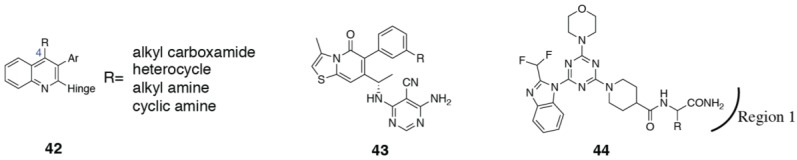
Structures of **42**–**44**.

Combinations that take advantage of both the tryptophan shelf and specificity pocket have also been explored. Bulky substitutions at the 4-position of a quinoline specificity pocket binding motif (**42**) explore the tryptophan shelf from the specificity pocket (PDB ID 5KAE) (Figure 8) [119,120]. A recent paper from GSK have described a novel binding mode for PI3Kδ inhibitors (see compound **29**), that involves a “conformational switch” of δTrp760 (Figure 5) [109]. The conformation of δTrp760 flips, such that the indole nitrogen is available to hydrogen bond with inhibitor functional groups that sit at the tryptophan shelf. Due to the anchoring hydrogen bonds to the equivalent tryptophan in PI3Kα and PI3Kγ (described in Section 3.1.2), this switch is likely to be energetically unfavorable in these isoforms. 

In addition to the specificity pocket, tryptophan shelf and interactions within the affinity pocket, interactions with the Region 1 non-conserved residues (also known as the ribose-binding region or hydrophobic region II) also influence selectivity. There are two key non-conserved residues in particular, which are accessible to inhibitors and are different between all four isoforms: δAsp832 (histidine, glutamate and threonine in p110α, β and γ, respectively) and δAsn836 (glutamine, aspartate and lysine in p110α, β and γ, respectively) (Figure 2c). Substitutions extending from the phenyl ring in idelalisib (**3**), or equivalent positions in different chemotypes, which extend toward Region 1, have been shown to influence selectivity [72,74,103]. Perry et al. [121] in their search for novel propeller-shaped chemotypes, also describe some SSAR of **43**, concentrating on substituents that extend toward Region 1 (Figure 8). Substitution of a primary or secondary amine maintains potency at PI3Kδ and increases selectivity against PI3Kα and PI3Kγ, but reduces selectivity against PI3Kβ. This can be explained by interactions with interactions with the Region 1 residue, δAsn836 and equivalent in the other isoforms. δAsn836 can accommodate the substitution, but a similar interaction is disfavored in PI3Kα by the longer sidechain in αGln859. The protonatable amine favors interaction with the negatively charged βAsp856, resulting in an increase in potency, but results in a repulsive interaction with γLys890, thus improving selectivity against PI3Kγ [121]. Our group has also published a series of PI3Kδ inhibitors targeting δAsn836 with a carboxamide motif (**44**, Figure 8) [122]. The inhibitor showed a 40x decrease in potency when δAsn836 was mutated to an aspartate (present in PI3Kβ at the equivalent position), confirming the interaction is important for isoform selectivity. 

### 3.3. PI3Kβ Selective Inhibitors

Many of the PI3Kβ selective inhibitors have also been shown to adopt a “propeller-shaped” binding mode. This may explain why many of the reported PI3Kβ selective inhibitors show only moderate selectivity against PI3Kδ. Very few of the inhibitors in this category have shown the exquisite selectivity common in PI3Kδ inhibitors, with most showing less than 100x selectivity against PI3Kδ. If, as has already been discussed, the specificity pocket is more easily accessible in both PI3Kβ and PI3Kδ, how do inhibitors distinguish between PI3Kβ and PI3Kδ? 

**Figure 9:**
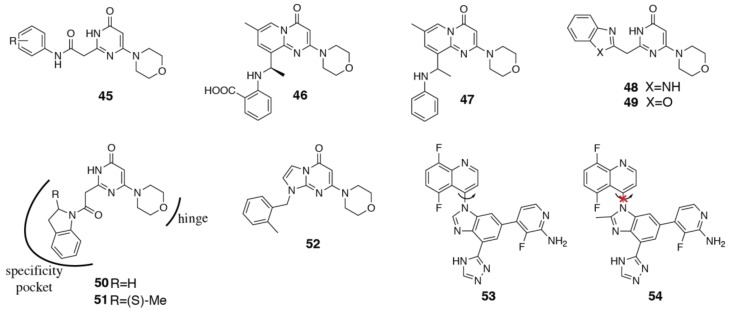
Structures of PI3Kβ selective inhibitors **45**–**54**. In the structure of **53**, there is free rotation around the bond highlighted with an arrow. The addition of a methyl group at the 2-position of the benzimidazole ring in **54** restricts rotation, thus forming two atropisomers.

One possibility is the lack of the tryptophan shelf in PI3Kβ. The equivalent residue to threonine is a lysine in PI3Kβ. The lysine sits on top of the tryptophan and provides an additional constraint to the specificity pocket in PI3Kβ [71]. The PI3Kβ specificity pocket may therefore favor smaller, more hydrophobic moieties. However, a well-positioned hydrogen bond donor may be able to pick up an interaction with the amino group of lysine and increase selectivity for PI3Kβ. Indeed, a survey of PI3Kβ selective inhibitors shows that the specificity pocket binding motifs are generally, but not exclusively, smaller than those found in PI3Kδ inhibitors. In a series of pyrimidone PI3Kβ inhibitors (**45**) with a phenyl specificity pocket motif, substitutions around the phenyl ring significantly affected the selectivity for PI3Kβ against the other isoforms [123]. *Meta* substitutions in particular seemed to improve both PI3Kβ potency and selectivity. The PI3Kβ inhibitor AZD6482/KIN193 (**46**) has an *ortho*-carboxyl substituent on the phenyl ring, however, the lack of a direct comparison in the same assay between this and TGX-221 (**47**) makes it unclear what effect this substitution has on the PI3Kβ/δ selectivity (Figure 9) [16,124].

Three related series of inhibitors have also been published, with either a benzimidazole (**48**), benzoxazole (**49**) or indoline (**50**, **51**) ring system as the specificity pocket binding moiety (Figure 9) [71,125]. The β/δ selectivity of these series is diminished compared with the original PI3Kβ selective inhibitor TGX-221, suggesting smaller substituents are favored by PI3Kβ [15]. However, in a study focusing on PI3Kβ/α selectivity, bulkier substituents were found to increase the selectivity over PI3Kα [126]. 

Optimal positioning of the specificity pocket binding motif may also have a greater influence on PI3Kβ/δ selectivity. In a series of imidazopyrimidone PI3Kβ inhibitors, the replacement of a 6,6-bicycle with a 6,5-fused ring system, in addition to shortening the linker to the specificity pocket binding motif (i.e., **52**, Figure 9) maintains potency at PI3Kβ, but also gains activity at PI3Kδ, thereby reducing the selectivity compared with TGX-221 [127,128]. An overlay of **52** docked into a PI3Kβ homology model shows a significant shift in the position of the phenyl ring in the pocket compared with TGX-221, which may account for the loss in selectivity [127]. In contrast, restricting flexibility of the specificity pocket binding motif and locking it in a propeller shape can increase selectivity. Chandrasekhar et al. [129] describe the development of a pair of atropisomeric compounds, one of which shows improved PI3Kβ potency and selectivity compared with the original analog with unrestricted rotation (**53**, **54**, Figure 9).

The effect of changes in the linker may also affect long-range interactions with the non-conserved residues in Region 1. An interesting study focused on improving the solubility of compound **50** found that a simple methyl substitution (**51**) increased selectivity for PI3Kβ over PI3Kδ from 7x to 20x (Figure 9). Crystal structures have been determined of **51** bound to both p110β (PDB ID 4BFR) and p110δ (PDB ID 4V0I), but yield no clues as to the rationalization of the selectivity, since the inhibitor makes no new interactions with the protein [71,130]. In an attempt to explain this striking difference, Robinson et al. [130] used the program, WaterMap, which computationally investigates solvation thermodynamics in the binding site of proteins with ligands bound. They proposed that differences in water networks in p110β and p110δ, caused by the non-conserved residues in Region 1 may explain the observed differences in selectivity [130]. This may also provide some rationale for other selectivity differences observed without direct interactions with the protein. For example, in a series of TGX derivatives, methylation of the aniline nitrogen dramatically improves potency at PI3Kα, γ and δ without affecting PI3Kβ, thus reducing selectivity [69]. The presence or absence of the hydrogen bond donor could have different effects on the water network of the various isoforms due to differences in Region 1. Interactions with Region 1 have been shown to be inconsequential with respect to the α/β selectivity of TGX-221 (**46**) [96], which is presumably more affected by accessing the specificity pocket, but may be more important in distinguishing between PI3Kβ and δ. 

**Figure 10:**
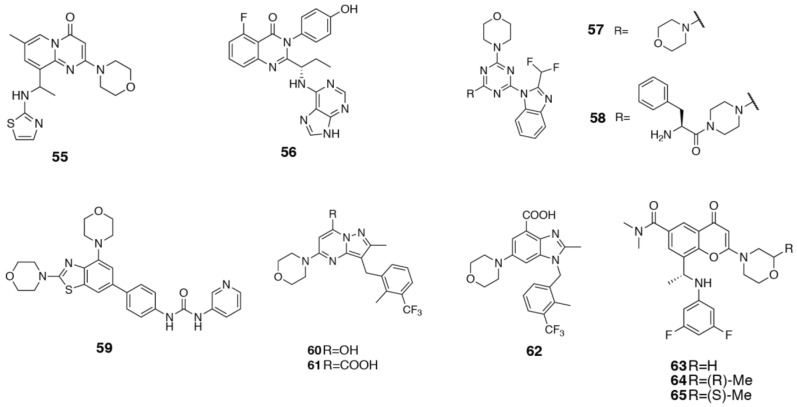
Structures of PI3Kβ selective inhibitors **55**–**65**.

The inhibitor BL140 (**55**), a derivative of TGX-221, with a thiazole replacing the phenyl to improve solubility, has similar PI3Kβ potency, but dramatically improved PI3Kβ/δ selectivity, from ~80x to >700x (Figure 10) [70]. The α/β selectivity is slightly reduced from 435x to 154x, but still maintains a good level. Unfortunately, no structural or modeling data is available to attempt to rationalize these changes. One possibility is that the inclusion of polar atoms in the ring alters the water networks in a way that favors PI3Kβ, as proposed by Robinson et al. [130]. However, it may also be possible that the smaller thiazole ring is not able to force open the specificity pocket, instead making direct interactions with the non-conserved residues of Region 1 and achieving its selectivity in this fashion. The lack of specificity pocket opening could thus explain the gain of potency at the α isoform. 

Other inhibitors have shown that interactions with the non-conserved residues of Region 1 play a significant role in determining selectivity. One study looked at rationally building in PI3Kβ inhibitory activity to the PI3Kδ inhibitor, idelalisib (**3**) [100]. The design strategy was to introduce hydrogen bond donors to the phenyl ring that could interact with βAsp862 (Asp856 follows the murine numbering). Quite strikingly, the introduction of a 4-hydroxy substituent (**56**) to the phenyl ring extending toward Region 1 increased PI3Kβ potency by more than 1000x, while maintaining PI3Kδ inhibition (Figure 10). This indicates the importance of Region 1 for modulating inhibitor selectivity. PI3Kβ selectivity was built into a pan-PI3K inhibitor starting point, ZSTK474 **57**, using a similar design strategy. The amine group of MIPS-9922 (**58**) was shown to achieve its selectivity via interaction with βAsp862 (Figure 10) [131,132]. Compound **59**, which has >150x selectivity for PI3Kβ, extends a morpholine substituent towards the same residue (Figure 10) [133]. Targeting this region proved essential in attempts to improve selectivity against PI3Kα to avoid potential side effects related to glucose regulation [134].

In many of the PI3Kβ selective inhibitors, the carbonyl sits in the affinity pocket, interacting with βTyr839 or βLys833 [71,98,123,125,126,134,135,136]. These do not extend as deeply into the affinity pocket as many of the PI3Kδ inhibitors [85]. This smaller binding motif may be important for PI3Kβ/δ selectivity in particular. In a series of dihydropyrazolo inhibitors, the replacement of a hydroxyl group (**60**) with the larger carboxyl group (**61**) reduces β/δ selectivity from 63x to only 8x (Figure 10) [137]. The clinical candidate, GSK2636771 (**62**) has a carboxyl group in the equivalent position. Unfortunately, an in-depth SSAR analysis has not been published, but presumably this contributes to the limited PI3Kβ/δ selectivity (10x) of **62** (Figure 10) [138]. 

Changes in the hinge-binding region can also surprisingly affect the selectivity, as was found in the case of PI3Kδ selective inhibitors. In the chromenone series described by Barlaam et al. [69], a 2-methyl makes little difference in PI3Kβ potency compared with the non-substituted morpholine **63** (Figure 10). The chirality of the substituent also is irrelevant for PI3Kβ. However, the R-methyl (**64**) improves PI3Kα and γ potency, while the S-methyl (**65**) reduces potency for PI3Kα, γ and δ, thus improving selectivity (Figure 10) [69]. 

### 3.4. PI3Kα Selective Inhibitors

**Figure 11:**
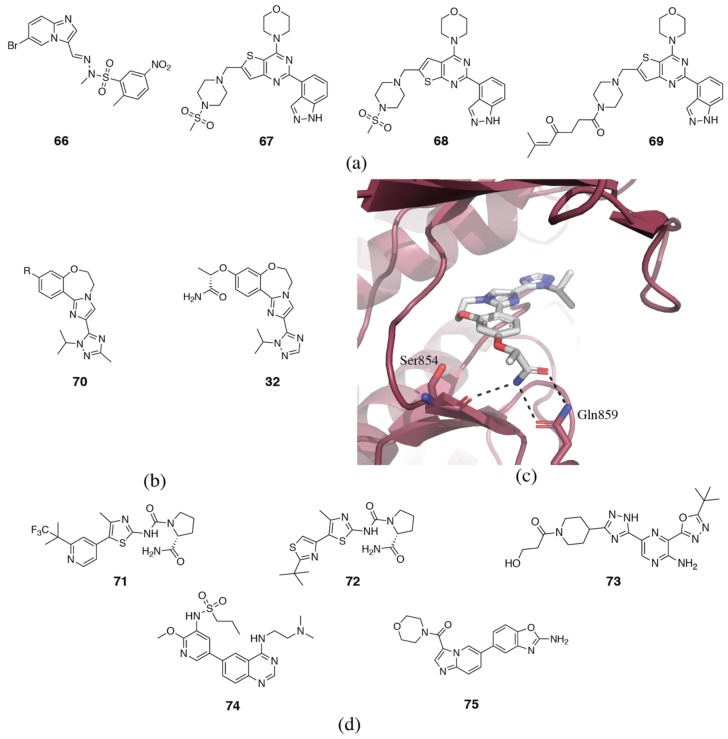
PI3Kα selective inhibitors. (**a**) Structures of **66**–**69**. (**b**) Structures of Genentech β-sparing (**70**) and α-selective inhibitors (**32**). (**c**) **32** in complex with p110α (raspberry, PDB ID 5DXT) showing Region 1 interactions with αGln859 and the backbone of αSer854. (**d**) Structures of **71**–**75**.

The first PI3Kα selective inhibitor to be described was PIK-75 (**66**, Figure 11a) [9,139]. It inhibits PI3Kα with an IC_50_ of 5.8 nM, and shows 13x selectivity over PI3Kγ, 88x selectivity over PI3Kδ and >200x selectivity over PI3Kβ [9]. SAR around the PIK75 scaffold has been carried out by multiple groups [139,140,141,142,143,144,145]. The bromo was shown to be important for overall potency [139,141,143,145]. The sulfonyl group appears to important for selectivity; replacement with a carbonyl retains potency at PI3Kα, but loses selectivity against the other isoforms [140,141]. The nitro group is important for potency and selectivity; replacement with an amino group increased potency at PI3Kα by 10x, but resulted in a loss of selectivity against PI3Kβ and PI3Kδ [140]. Changing the N-methyl substitution to an ethyl showed conflicting effects on PI3Kα selectivity; Schmidt-Kittler et al. [140] showed an overall improvement in PI3Kα selectivity, while the 6-cyanoimidazopyridine analogs of Kendall et al. [141] showed a loss of selectivity over PI3Kδ with an N-ethyl substitution. Substitutions around the phenyl ring appear to be important for selectivity [140]. Replacing the 2-methyl with a 2-chloro was equipotent at PI3Kα, but lost PI3Kδ selectivity [140]. A 4-fluoro substitution increased selectivity over PI3Kγ, but lost selectivity over PI3Kδ, while a 4-glycylphenylalanine gave 600x selectivity against PI3Kβ, 60x against PI3Kγ and 100x against PI3Kδ [140]. Despite the extensive SAR available, the absence of a co-crystal structure with any isoform has limited rationalization of the selectivity. At least four groups have published different computational predictions of binding modes [146,147,148,149]. Site-directed mutagenesis was able to test each of these hypotheses experimentally and found that PI3Kα specific interactions with αHis855 and αSer773 in Regions 1 and 2, respectively, were important for the isoform selectivity [97]. This corresponds most closely with the model proposed by Frédérick and Denny [146], where the imidazopyridine ring interacts with the hinge region, the bromine extends into the affinity pocket, the sulfonyl interacts with αHis855, and the nitro group interacts with αSer773. The latter interaction would explain the loss of selectivity against PI3Kβ and δ when the nitro is substituted to an amino group, as both these isoforms have an aspartate at this position. PI3Kγ has an alanine, and its potency is unaffected by the substitution [140]. The model also suggests hydrophobic interactions with αTrp780 and an interaction between the nitro group and αArg770, which occludes the tryptophan shelf in PI3Kα [146]. However, mutation of αArg770 to an alanine did not significantly affect the IC_50_ of PIK75, suggesting this interaction is less important than that with αSer773 [97]. Subsequent to the modeling and mutagenesis studies, a crystal structure of PIK75 in complex with the protein kinase GSK-3β was determined (PDB ID 6GN1) [150]. Similar to Frédérick and Denny’s [146] model, the imidazopyridine ring binds at the hinge region, with the bromine extending into the affinity pocket. The nitro group, however, rather than interacting with residues on the P-loop, extends into the affinity pocket and forms an intramolecular interaction with the bromo group [150]. This binding mode would not seem to correspond to the mutagenesis or available SAR, and it seems likely that PIK75 adopts an alternative binding mode in PI3K. 

Genentech have also reported the importance of the P-loop residues in PI3Kα selectivity [151]. Modification of the core of their pan-PI3K inhibitor, GDC-0941 (**67**), to an isomeric thiophene (**68**), increased PI3Kα/β selectivity from 10x to 100x (Figure 11a). They postulate that this is a conformational effect and influences the positioning of the sulfonyl and its potential for hydrogen bonding with the non-conserved αArg770 and the backbone of αSer773 [151]. Also starting from GDC-0941, Nacht et al. [152] describe the design of a targeted PI3Kα selective covalent inhibitor (**69**, Figure 11a). They extend an enone from the piperidine ring to react with the PI3Kα-specific residue, αCys862, thus imparting selectivity and forming a covalent linkage (PDB ID 3ZIM). In the other isoforms, the residue at this position is leucine in PI3Kβ and PI3Kδ and glutamine in PI3Kγ, removing the possibility of irreversible inhibition.

The most effective region of the binding site to target for PI3Kα selectivity has been found to be Region 1. In the development of benzoxazepin β-sparing PI3K inhibitors, Genentech describes the importance of the moiety extending towards Region 1 for modulating selectivity (**70**, Figure 11b) [151,153,154]. In particular, they found that positively charged substituents favor PI3Kβ, due to the negatively charged βGlu858 and βAsp862, while bulky, aromatic, uncharged substituents show favorable PI3Kα/β selectivity [153]. This was an unexpected finding, as the uncharged substituents do not seem able to form hydrogen bonds with the non-conserved residues αHis855 and αGln859. Staben et al. [153] propose that the alternative conformation of βTrp777 observed in the PI3Kβ crystal structure (PDB ID 2Y3A) may contribute to this observed selectivity. In PI3Kα, αTrp780 is anchored by a hydrogen bond from the indole nitrogen to αGlu798, and a similar hydrogen bond is observed with γGlu814 in PI3Kγ. A valine residue at the equivalent position in PI3Kβ not only does not provide a hydrogen bonding partner, but may also disfavor a similar orientation of the tryptophan ring. In this case, one potential explanation is that the steric bulk of substituents are not as well accommodated by the alternative tryptophan conformation in PI3Kβ [153]. 

These studies eventually culminated in the discovery of GDC-0326 (**32**), a PI3Kα selective inhibitor (Figure 11b) [67]. The structures of GDC-0326 bound to both PI3Kα (PDB ID 5DXT) and PI3Kδ (PDB ID 5DXU) have been determined [67]. In PI3Kα, the carboxamide in GDC-0326 forms complementary interactions with αGln859, and a hydrogen bond to the backbone carbonyl of αSer854 (Figure 11c). In PI3Kδ, the inhibitor forms two hydrogen bonds with the backbone carbonyl of αSer831, and with the side chain of δAsp832. The serine hydrogen bond is at an increased distance and the aspartate hydrogen bond appears to be at a suboptimal angle, providing some rationale for the observed selectivity [67].

Novartis reported the optimization of a series of 2-aminothiazole inhibitors which also target αGln859 [65,155,156]. Before the determination of the crystal structure of the clinical candidate, BYL719 (**71**) in PI3Kα (PDB ID 4JPS), modeling [157] and site-directed mutagenesis [158] studies with A-66 (**72**), a related 2-aminothiazole analog, strongly implicated an interaction with αGln859 as determining the exquisite PI3Kα selectivity of these compounds (Figure 11d). The subsequent determination of the crystal structure confirmed these studies [65].

Astra-Zeneca have also published their efforts toward developing a PI3Kα inhibitor, predominantly targeted towards optimizing interactions with αGln859 (**73**, Figure 11d) [159,160,161]. Rather than a carboxamide moiety, their compounds exhibit a pyrazole ring that is thought to interact with αGln859. Fan et al. [162] have also reported a series of PI3Kα selective inhibitors (**74**) targeting αGln859, via a bidentate interaction between a methoxy group and sulfonamide nitrogen, both extending from a substituted pyridine ring (Figure 11d).

In PI3Kβ and δ selective inhibitors, substitutions in the affinity pocket have been shown to modulate selectivity. This appears to be less important in PI3Kα selective inhibitors, with affinity pocket binding motifs contributing more to potency than isoform selectivity [153,154]. 

Intellikine’s inhibitor, MLN1117 (**75**), has none of the above mentioned features, but still exhibits greater than 125x selectivity for PI3Kα (Figure 11d) [66]. Unfortunately, no structure or SSAR data has been published to allow for an understanding of the molecular determinants of this selectivity.

### 3.5. PI3Kγ Selective Inhibitors

**Figure 12:**
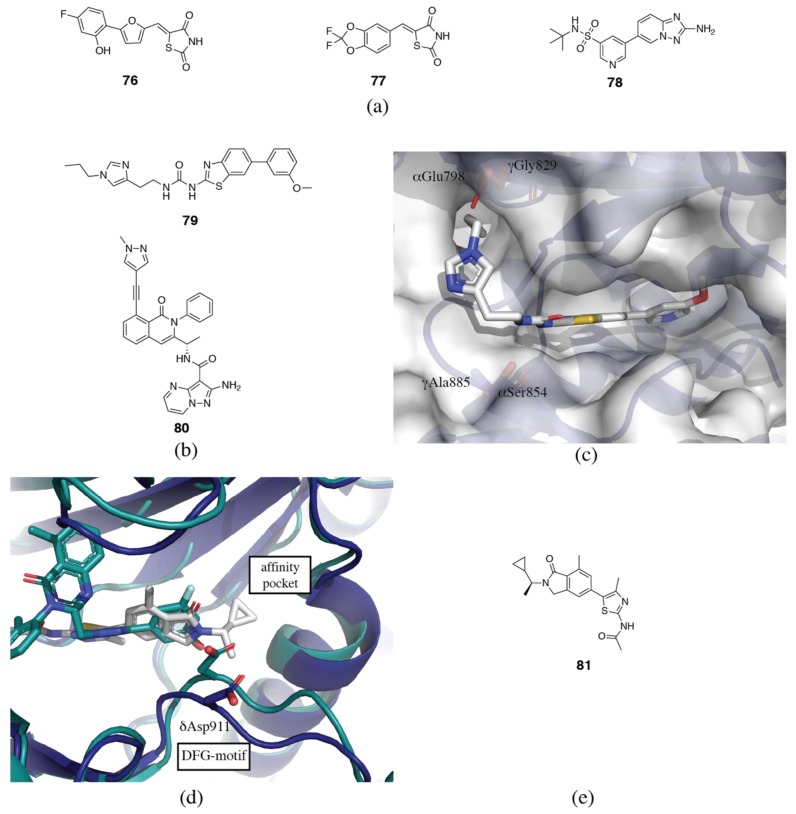
PI3Kγ selective inhibitors. (**a**) Structures of **76**–**78**. (**b**) Structures of **79**–**80**. (**c**) The hydrophobic binding cleft in p110γ (PDB ID 4PS3). The structure of p110γ bound to **79** is shown in dark blue cartoon and light gray surface representation. An overlay with p110α (raspberry, PDB ID 4JPS) highlights how αGlu798 obscures the binding cleft. The cartoon of p110α has been hidden for clarity. (**d**) The “alkyl-push”. The structure of **81** bound to p110δ (dark blue, PDB ID 6FTN). The cyclopropyl tail of **81** (shown in white) extends deeper into the affinity pocket than previously observed. The structure of **1** bound to p110δ (teal, PDB ID 2WXG) has been shown as a comparison for typical affinity pocket binding moieties. A shift in the loop containing the DFG-motif and helix at the back of the affinity pocket can be observed. (**e**) Structure of **81**.

The structural basis of selectivity for some of the earliest PI3Kγ selective inhibitors is difficult to rationalize. The first series of PI3Kγ inhibitors to be published were based on the rhodanine or thiazolidinedione scaffold [163,164]. Crystal structures of several family members reveal that the rhodanine portion binds in the affinity pocket, while the variable motif binds to the hinge region [163,164]. No other regions of the binding pocket are engaged. As we have discussed, the affinity pocket contains only conserved residues, but has been shown to vary in flexibility and water networks, so motifs binding in this pocket do appear to modulate isoform selectivity. The rhodanine series of inhibitors generally show good selectivity over PI3Kβ and δ, but only moderate PI3Kα selectivity. Interestingly, the two inhibitors that show the best PI3Kγ/α selectivity (30x), AS-252424 (**76**) and AS-604850 (**77**) (PDB ID 2A4Z), both have hinge-binders that extend towards γAla885, which is a serine in all the other isoforms (Figure 12a) [163,164]. In all the other isoforms, this residue is a serine and forms a hydrogen bond with the backbone of δVal828 [165]. Disruption of this hydrogen bond in PI3Kα, β and δ without sufficient compensation elsewhere in the binding site may contribute to the selectivity observed in these inhibitors. 

Cellzome published a series of triazolopyridines as PI3Kγ inhibitors (**78**, Figure 12a) (PDB ID 4AOF) [166,167,168]. The amino-triazolopyridine forms a bidentate interaction with the hinge region. The derivatization focused on the affinity pocket binding portion. As has previously been noted, affinity pocket binding motifs have a surprisingly important effect on selectivity. Substituents at the 3-position of the phenyl ring were preferred, generally showing submicromolar potency, while selectivity over PI3Kα and δ varied from as little as 4x to as much as 500x [166]. Subsequent development of these compounds focused on heterocyclic and glycinamide ureas in place of the free amino group for PI3Kγ/δ dual-selective inhibitors [169].

In the course of development of 4-aza-isoindolinone PI3Kγ selective inhibitors (**79**, Figure 12b), Vertex Pharmaceuticals discovered a novel hydrophobic binding cleft adjacent to the hinge region, which confers selectivity for PI3Kγ (Figure 12c) [165,170,171,172]. The binding cleft is formed by the combination of two key non-conserved residues, γAla885 (serine in all other isoforms) and γGly829 (glutamate in PI3Kα, glycine in PI3Kβ and δ) that allow access to this area of the binding site (PDB ID 4PS3) [165]. In particular, bulky protrusions into the hydrophobic cleft give good selectivity over PI3Kα, due to unfavorable interactions with the glutamate residue.

IPI-549 (**80**) which is currently in clinical trials for advanced solid tumors, is an exquisitely selective propeller-shaped PI3Kγ inhibitor (Figure 12b) [57]. Starting with an 8-chloroisoquinolinone core as a specificity pocket binding motif, commonly found in PI3Kδ inhibitors, the hinge region was explored. A pyrazolopyrimidine-2-amine hinge-binding motif, which resembles the triazolopyridines in the Cellzome inhibitors, showed 10x selectivity for PI3Kγ over PI3Kδ. Further selectivity was achieved via substituted alkynes at the 8-position of the isoquinolinone core. PI3Kδ selective inhibitors with 8-alkynyl substituents were docked and found to interact with δGln748 (γLys800) or δLys712 (γIle764) (Figure 3a) [119]. These are both non-conserved residues. It is possible that the N-methylpyrazole group interacts with γLys800 or γIle764, or another non-conserved residue in this area.

The existence of PI3Kβ, γ and δ selective propeller-shaped inhibitors brings into question the initial hypothesis that the specificity pocket conveys PI3Kδ selectivity. It appears to be much more complicated, with selectivity being determined by an amalgamation of interactions within the binding site. It could be that the specificity pocket is most easily accessible by PI3Kδ, followed by PI3Kβ and γ, and finally by PI3Kα. Despite the abundance of crystallographic data available, the isoform specificity of the specificity pocket remains unclear.

A recently published series of aminothiazole PI3Kγ inhibitors (**81**) with an N-isopropylcyclopropane tail that inserts deep into the affinity pocket shows remarkable PI3Kγ selectivity (PDB ID 6FTN) (Figure 12d,e) [173]. Removal of the N-alkyl tail shows a dramatic 100x drop in PI3Kγ potency, while increasing potency at PI3Kα and β by more than 10x. PI3Kδ potency remained essentially unchanged. The N-alkyl tail extends further into the affinity pocket than other reported inhibitors, inducing the formation of a new pocket by a so-called “alkyl-push” and this may be a key feature that contributes to the PI3Kγ selectivity [49,173]. Surprisingly, the inhibitor did not successfully co-crystallize in PI3Kγ, and the published structure is in complex with PI3Kδ, which may not reveal all the relevant structural details for understanding the selectivity. The authors hypothesize that PI3Kγ has greater flexibility that the other isoforms.

### 3.6. Rational and Irrational Isoform Selectivity

**Figure 13:**
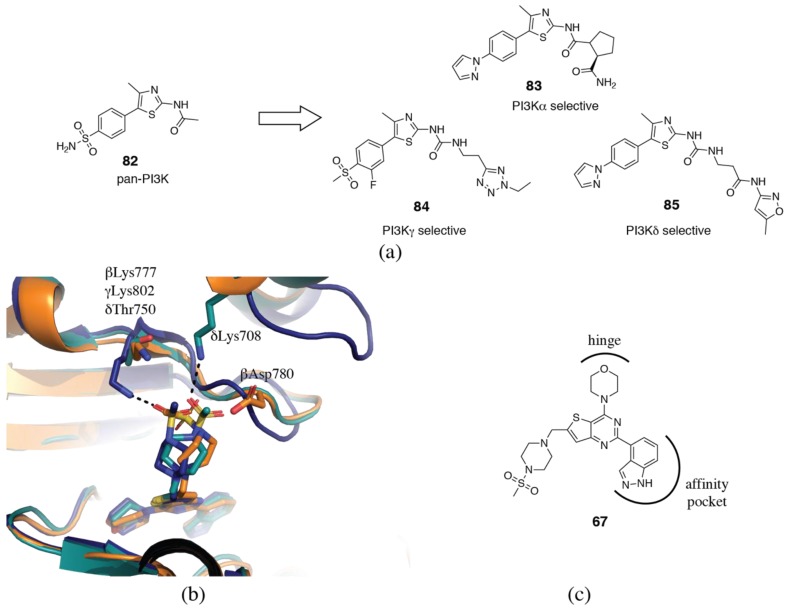
Rational and irrational selectivity. (**a**) The rational design of isoform selective inhibitors **83**-**85** from a pan-PI3K core (**82**). (**b**) GDC-0941 (**67**) bound to p110β (orange, PDB ID 2Y3A), p110γ (dark blue, PDB ID 3DBS) and p110δ (teal, PDB ID 2WXP). The sulfonyl group shifts conformation depending on which non-conserved residues are present. Hydrogen bonds have been shown in black dashed lines. (**c**) The structure of GDC-0941 (**67**).

An interesting case study published by Bruce et al. [155] presents the anticipated conclusion from all of this knowledge: the ability to rationally design individual PI3Kα, γ and δ selective inhibitors starting from a pan-PI3K core structure (**82**, Figure 13a). By introducing a prolinyl-carboxamide extending towards αGln859, they are able to introduce PI3Kα selectivity to the core structure (**83**). By introducing long alkyl chains, they are able to target the hydrophobic binding cleft near the hinge region and introduce PI3Kγ selectivity (**84**). Finally, a PI3Kδ inhibitor is developed with an amide linked to an isoxazole (**85**). The rationale for the PI3Kδ selectivity is less clear, perhaps it interacts with a non-conserved residue of Region 1 or can access the tryptophan shelf.

Another interesting case study, this time in the irrationality of inhibitor selectivity, is the Genentech pan-PI3K inhibitor, GDC-0941 (**67**, Figure 13b,c) [174]. It has been crystallized in complex with PI3Kβ, γ and δ (PDB ID 2Y3A, 3DBS, 2WXP, respectively) [85,86,174]. In PI3Kγ, the methyl sulfonyl group interacts with the non-conserved γLys802 (Figure 13b) [174]. In PI3Kδ, where this lysine is replaced by a threonine, the sulfonyl group has shifted, and now interacts with δLys708 (αGln728, βArg729, γSer760) and the backbone of δAsp753 [85]. In PI3Kβ, the piperidinesulfonamide adopts yet another conformation. Note however, that care should be taken when interpreting hydrogen bonds in the PI3Kβ structure due to the low resolution (3.5 Å). Hydrogen bonds with βLys777 (equivalent to γLys802) and βAsp780 (equivalent to δAsp753) are possible, but electron density is missing for these side-chains. This highlights the role of inhibitor flexibility allowing the adoption of multiple conformations in the binding site and resulting in pan-PI3K inhibition despite interaction with non-conserved residues. It also emphasizes the complex nature of isoform selectivity and the difficulties associated with rational design.

### 3.7. PI3Kα Oncogenic Mutant Selectivity

One aspect of selectivity that is seldom discussed is that of selectivity for the PI3Kα hotspot mutants, αGlu542Lys, αGlu545Lys and αHis1047Arg over the wild-type PI3Kα. Given the essential functions of PI3Kα, selectively inhibiting the relevant oncogenic mutants may widen the therapeutic window and reduce adverse effects of treatment [175,176]. Wang et al. [177] describe their attempts via high throughput screening to develop an inhibitor selective for His1047Arg-PI3Kα. The identified hits have IC_50_ values in the micromolar range, and although some show differential inhibition between the mutant and wild-type forms, they generally favor the wild-type enzyme. Sabbah et al. [178] identified a series of *N*-phenyl 4-hydroxy-2-quinolone-3-carboxamides that display 10–20x selectivity for a His1047Arg colon cancer cell line over the isogenic WT cell line. Unfortunately, they do not present data determined from assaying with purified enzyme. Gkeka et al. [179] explore the potential of a non-ATP pocket that was identified in a PI3Kα crystal structure for selective inhibition of the His1047Arg mutant [88]. Ultimately, they conclude that this binding site is unlikely to yield productive allosteric inhibitors. Our lab has explored the use of fragment-based screening to identify novel binding sites that have the potential for allosteric inhibition [180]. One site that was identified is in the region of the phosphopeptide binding site and coincides with the position of two of the three hotspot mutations, αGlu542Lys and αGlu545Lys, suggesting the potential for development of oncogenic mutant inhibitors at this site.

## 4. Conclusions

The highly homologous ATP-binding site of Class I PI3Ks has yielded more opportunities for isoform selective inhibitors than was originally anticipated. Key regions of the binding site: the specificity pocket, tryptophan shelf, hydrophobic binding cleft, “alkyl-push” and Region 1 have been identified that allow for a degree of rational design to be attempted. However, there are still key aspects to inhibitor selectivity that remain unclear. How exactly does the specificity pocket contribute to selectivity? How do hinge and affinity binding motifs contribute to selectivity, when these regions are made up of conserved residues? Isoform selectivity, and inhibitor binding in general, results from a complex amalgamation of interactions throughout the binding site, influenced by protein and inhibitor conformational flexibility. 

## Figures and Tables

**Table 1:** Single and dual-isoform selective phosphatidylinositol 3-kinase (PI3K) inhibitors approved for use or under clinical evaluation.

Inhibitor	Other Names	Target	PDB ID (isoform)	IC_50_ (nM)			
				PI3Kα	PI3Kβ	PI3Kγ	PI3Kδ
Alpelisib [65]	NVP-BYL719	α	4JPS (α)	5	1200	250	290
Serabelisib [66]	MLN1117; INK1117; TAK-117	α	-	15	4500	1900	13,900
GDC-0326 [67]		α	5DXT (α)	0.2 ^1^	26.6	4	10.2
CYH33 [68]		α	-	nd	nd	nd	nd
AZD8186 [69]		β	-	35	4	675	12
GSK2636771 [70]		β	-	35,400	20	nd	40
SAR260301 [71]		β	4BFR (β)	1539	23	10,000	468
IPI-549 [57]		γ	-	3200	3500	16	>8400
AMG319 [72]		δ	4WWN (γ)	33,000	2700	850	18
GSK2292767 [73]		δ	5AE9 (δ)	501	630	501	0.079
Idelalisib [74]	CAL-101, GS-1101	δ	4XE0 (δ)	8600	4000	2100	19
Leniolisib [75]	CDZ173	δ	5O83 (δ)	244	424	2230	11
Nemiralisib [73]	GSK2269557	δ	5AE8 (δ)	5011	1584	6309	0.12
Umbralisib [49]	TGR-1202	δ	-	>1400	>756	>120	14
Parsaclisib [49]	INCB050465	δ	-	nd	nd	nd	1
Duvelisib [76]	IPI-145	γ/δ	-	1602	85	27	2.5
RV1729 [49]		γ/δ	-	192	-	25	12
RV6153 [49]		γ/δ	-	14,000	5000	28	2.5
Tenalisib [49]	RP6530	γ/δ	-	10,000	4000	33	25

^1^ Reported data is Ki_app_ rather than IC_50_ (half-maximal inhibitory concentration). nd—not disclosed.

**Table 2:**
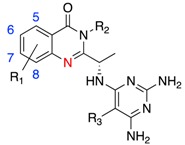
Effects of modifications around a quinoxalinone core on potency and selectivity. Substitutions modify the basicity of N1, which is colored in red.

Compound	R1	R2	R3	IC_50_ (nM)	Fold Selectivity		
				PI3Kδ	α/δ	β/δ	γ/δ
**8** ^1^	5-Cl	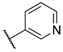	CN	0.6	1716	166	120
**9** ^1^	8-Cl			6	517	65	183
**10** ^1^	5,8-Cl			0.7	1857	108	470
**11** ^1^	5-F,8-Cl			0.4	1925	115	127
**12** ^2^	6-F	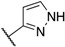	Cl	9.8	8	4	238
**13** ^2^	6-F,8-Cl			5.3	109	1.5	>357
**14** ^3^	H	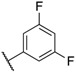	CN	15	530	290	74
**15** ^3^	6-F			4.8	600	520	160
**16** ^3^	5-Cl, 6-F			2.9	780	470	74

^1^ data taken from [99], ^2^ data taken from [100], ^3^ data taken from [101].
